# Commentary on "Preliminary Species Hypotheses" in Entomological Taxonomy: A Global Data and FAIR Infrastructure Perspective

**DOI:** 10.3897/BDJ.13.e141562

**Published:** 2025-02-10

**Authors:** Sharif Islam

**Affiliations:** 1 Naturalis Biodiversity Center, Leiden, Netherlands Naturalis Biodiversity Center Leiden Netherlands; 2 DiSSCo, Leiden, Netherlands DiSSCo Leiden Netherlands

**Keywords:** taxonomy, species, interoperability, FAIR, data integration, open source

## Abstract

What if early taxonomic findings were treated like preprints, open to iterative improvement or managed with practices from the open-source community, such as Git branching, merging and patch management? Prompted by Buckley's article *Charting a Future for Entomological Taxonomy in New Zealand* (2024), this commentary explores these possibilities in the context of biodiversity informatics. In response to the need for rapid, scalable biodiversity monitoring, Buckley introduces preliminary species hypotheses (PSH) as a bridge between quick identification tools and the rigorous Linnaean system, leveraging DNA barcoding and AI-assisted image recognition to produce provisional classifications that can later be validated. Expanding on Buckley’s framework, this commentary emphasises the critical role of data linking, versioning and integration to support evolving taxonomic data. Borrowing from software and open-source practices, I explore the idea of managing PSH with an infrastructure that treats each taxonomic update as a versioned "commit", which can be tracked, refined and integrated over time. Drawing insights from FAIR (Findable, Accessible, Interoperable, Reusable) principles and Digital Extended Specimens, I identify infrastructure requirements for PSH, including robust data standards, persistent identifiers and interoperability to support global biodiversity repositories. Additionally, Taxonomic Data Objects offer a model for dynamically integrating PSH into adaptable taxonomies that can evolve with new data and tools. By positioning PSH within an open, infrastructure-focused framework, this commentary advocates for scalable, hypothesis-driven biodiversity data that meets modern conservation needs, bridging traditional and emerging practices in taxonomy.

## Introduction

In *Charting a Future for Entomological Taxonomy in New Zealand*, published in the journal *New Zealand Entomologist*, T.R. Buckley (2024) proposes the concept of preliminary species hypotheses (PSH) as a way to bridge the gap between the need for rapid species identification and the rigorous Linnaean taxonomy. Buckley argues that PSH can address biodiversity monitoring needs by utilising output of rapid identification tools -- such as DNA barcoding and AI-assisted image recognition - as provisional classifications that serve as an intermediate stage before formal taxonomic classification. Although based in New Zealand and focused on entomology, this proposal has implications for other regions and fields within taxonomy and biodiversity research.

Buckley's proposal envisions scalable, hypothesis-driven biodiversity data that can evolve as new information emerges. Inspired by this approach, we might ask: What if early taxonomic findings were treated like preprints (Verma and Detsky 2020) - open to iterative improvement? Or managed through practices adapted from open-source software development, such as Git branching, merging and patch management, where each PSH acts as a versioned "commit"? While this iterative process echoes how taxonomic science has traditionally progressed, this approach could offer a flexible framework for tracking and refining taxonomic data over time.

In this commentary, I explore the data linking and integration infrastructure required to support Buckley’s vision, emphasising how PSH fits within the broader framework of the Species Hypotheses (SH) and Taxon Hypotheses (TH) paradigm. These concepts also align with evolving Biodiversity Information Standards (TDWG) standards like the Taxon Concept Schema (TCS), which separates Taxon Concepts from Taxon Names to enhance data interoperability (Klazenga and Liljeblad 2024). Based on these conceptual frameworks, I explore the data linking and integration infrastructure required to support Buckley's vision, drawing on knowledge infrastructure studies such as Christina Borgman's work on data systems (Borgman and Brand 2024) and Sterner et al.'s pluralistic framework for biodiversity data sharing (Sterner et al. 2023). I also consider recent proposals, such as Digital Extended Specimens (Hardisty et al. 2022) and Taxonomic Data Objects (Upham and Poelen 2024), as potential models for integrating PSH and other hypotheses-driven insights (both from molecular- and media-based multimodal workflow) as data products within global biodiversity infrastructures. This infrastructure-based approach can help sustain taxonomy's relevance in conservation and research. My focus is not on assessing the scientific rigour of PSH, but rather on the data linking and integration strategies that could underpin its implementation, offering a scalable pathway for the evolution of taxonomic knowledge.

## Summary of paper

Buckley’s proposal introduces PSH as a practical and flexible way to address the gap between rapid biodiversity identification needs and the more formal Linnean classification system. The paper presents the proposal with a historical background of entomological taxonomy in New Zealand, discussing the reasons for declining taxonomy funding and the importance of maintaining scientific rigour. For relevance, the summary here highlights the key concepts of PSH.

This provisional approach of PSH aligns well with similar concepts, such as Operational Taxonomic Units (OTUs) in DNA barcoding, which serve as proxies to categorise unidentified taxa for integration across different biodiversity datasets and use cases. According to Buckley, OTUs are typically molecular-based groupings (often not derived from DNA-sequenced specimens, particularly for environmental DNA data). As Buckley (2024):9 states:

“…it is difficult to reconcile these OTUs [OTUs that are not derived from DNA sequencing specimens and do not have a physical reference specimen] with other types of character data. From a hypothesis testing perspective, these OTUs can also be considered ‘preliminary species hypotheses’, but with a weaker degree of support than from specimen-based DNA sequencing approaches (as outlined earlier). This approach will require a large-scale eDNA survey of New Zealand, focusing on the sampling of soil, water, air and insect trap residues. Achieving this goal would also be a 5- to 10-year project with a moderate financial investment. The output would be a comprehensive database of OTUs that, over time, could be connected to described species or to DNA sequences obtained from individual specimens".

In contrast, PSH are structured as an intermediate classification that is less formal than Linnaean taxonomy, but aspires to achieve it over time. Unlike OTUs, PSH are not simply molecular clusters; they are hypotheses that can later be validated and incorporated into formal taxonomy as additional data become available. Buckley also reminds us in the paper that similar methods are commonly used in fields of mycology (Kõljalg et al. 2013) and bacteriology. While OTUs offer a rapid and flexible tool for biodiversity estimation, PSH are designed to be a step closer to formal species recognition, enabling hypothesis-driven research and prioritisation without bypassing rigorous taxonomic standards entirely:

“The goal is not to replace the Linnean system, or to lower its scientific robustness, but to provide a framework for describing biodiversity more quickly than Linnean taxonomy can. DNA data can characterise lineages that, in turn, can be considered as ‘preliminary species hypotheses’. These hypothesised species can be tested, verified and described by taxonomists later if resources become available. In the meantime, these hypothesised species can be used as a basis in downstream conservation actions or ecological studies that require biodiversity to be divided into scientifically meaningful entities. However, it must be remembered that these hypothesised species have not been subject to robust testing and, therefore, any downstream inference will not be as reliable as that from a fully revised taxon” (Buckley 2024:8).

Furthermore, the robustness and benefit of the hypothesis-driven and iterative approach come not just from a single data type, but from integrating a variety of data types. For instance, combining molecular-based methods and multi-modal AI techniques can significantly reduce uncertainties in the inference of observations:

"If we want robust species hypotheses, then large numbers of characters will continue to be needed. There are technologies emerging that promise to greatly increase the rate of data collection without sacrificing scientific robustness. The approach adopting these technologies is known as *large-scale integrative taxonomy* (Hartop et al. 2022; Salili-James et al. 2023; Karbstein et al. 2024). Briefly, this approach comprises two steps. First, high throughput methods are used to collect character data and perform a provisional grouping of specimens into putative species. Second, another character type, with a high *a priori* probability of being incongruent with the first character set, is used to test those putative species (Hartop et al. 2022). A key feature is the use of technology to accelerate the rate and scale of data collection" (Buckley 2024:7).

The demand for taxonomic information for a variety of use cases (such as environmental monitoring and biosecurity) is rising, making traditional insect sampling and identification methods increasingly impractical, especially amidst a shortage of experts. New technologies, including DNA barcoding, eDNA for community assessments and automated image recognition, offer promising alternatives that can democratise species identification. Automated image recognition, in particular, enables non-specialists to identify insects, making taxonomy more accessible. However, according to Buckley, successful adoption of these tools requires extensive digitisation of specimen records and integration with images, DNA sequences and geo-referenced data.

## Key Terms, Definitions and Alignment with Existing Concepts

The practice of taxonomy and nomenclature deals with different concepts and terms beyond naming species (see Favret (2024) for 5 'D's of taxonomy: delimitation, diagnosis, description, determintation and discovery) where the aspect of testable hypotheses intersects all of these concepts. While detailing every aspect is beyond the scope of this commentary, this section defines key terms and situates them within the evolving landscape of biodiversity informatics. The following concepts are briefly stated here to facilitate the discussion and lay the foundation for understanding how PSH can integrate into taxonomic workflows and biodiversity data infrastructures.

**Barcode Index Numbers (BINs)**: BINs are molecular-based clusters derived from DNA barcoding, primarily serving as proxies for species identification using genetic divergence thresholds. BINs are similar to OTUs, but are specific to DNA barcoding. Unlike OTUs, which are often used as an intermediate step requiring further species-level identification, BINs are dynamic and the boundaries of what sequences can be associated with a particular BIN can change with new sampling data (Ratnasingham and Hebert 2013; Lue et al. 2022) and one BIN can cover more than one taxon (Huemer and Mutanen 2022).

**Species Hypotheses (SH)**: SH is the main building block of UNITE (a database and sequence management environment centred on the eukaryotic nuclear ribosomal ITS region) which groups similar sequences into provisional species-level clusters typically comprising two or more sequences to avoid excessive inflation (Kõljalg et al. 2013). Representative sequences for each SH are chosen through consensus computation or expert designation. These SHs, along with their representative sequences and annotations, are made available as reference datasets. Buckley's paper discusses SH used in mycology and explores how entomology can adapt similar ideas. This discussion also opens up the possibility of integrating SH concepts for broader use beyond mycology and zoology, not necessarily limited to DNA-based identification methods.

**Taxon Hypotheses (TH) paradigm**: Expanding on the SH concept, Kõljalg et al. (2020) introduces the TH paradigm that represents a framework for linking sequence-based identifications to taxonomic concepts. By assigning Digital Object Identifiers (DOIs) to these hypotheses, THs enable transparent and reproducible connections between molecular data and taxonomic classifications. Kõljalg et al. (2020) also highlights that, while molecular data are becoming increasingly common, differences in sampling, genetic markers and analytical methods often lead to competing and sometimes conflicting classifications. The reference datasets and DOIs provided by UNITE offer a unique reference point that remains consistent even as underlying data and conclusions evolve. This system allows users to reference the data enabling modifications and augmentations, while preserving original versions.

All of these frameworks have one thing in common: they acknowledge the dynamic and "preliminary" nature of initial insights into species identification. Thus, PSH or SH could emerge as a new "data type" that can be used not just in mycology or zoology, but across domains. Furthermore, this approach supports integrative methods that apply multiple types of characters, leading to robust hypothesis tests and, therefore, greater confidence in the acceptance or rejection of a species hypothesis.

Recent discussions (see Karbstein et al. (2024)) on species delimitation and AI also underscore the importance of incorporating multiple data types and frameworks such as unified species concept, morphological and phylogenetic (genetic relationships and shared ancestry) and DNA clustering methods that are going towards a more integrative approach (genetics/genomics + morphology + ecology). AI-based identification methods, including multimodal approaches involving sound and vision, are also becoming increasingly prevalent (Wäldchen and Mäder 2018; Yang et al. 2021). Each approach has limitations; thus, integrative approaches that combine multiple lines of evidence align with the dynamic nature of species hypotheses.

By situating PSH, SH, TH, BINs and OTUs within a unified conceptual framework, this commentary underscores the value of treating species hypotheses as dynamic, evolving data objects. Each concept - BINs, OTUs, SH, TH and PSH - has distinct origins rooted in specific fields, such as molecular biology, fungal taxonomy and entomology. These approaches complement the Linnaean classification by integrating preliminary taxonomic data into an iterative process that refines and validates hypotheses over time. Expanding their application to encompass diverse data types will enhance their utility across taxonomic domains. A holistic and integrative approach supports the iterative refinement of taxonomies while balancing the need for rapid discovery with the production of robust, high-quality data.

## The role of infrastructures

Following the summary of Buckley’s PSH proposal, it becomes clear that data integration and linking will be an important aspect and, thus, the successful implementation and sustainability of PSH require a robust digital infrastructure. This infrastructure not only enables data sharing, but also supports the evolution of taxonomic knowledge in a scalable and accessible way. The PSH model is comparable to preprints in scholarly publishing: it provides a way to make new insights accessible, citable and linkable, even if they require further refinement and validation. When viewed through the lens of the Digital Extended Specimen (DES) paradigm (Hardisty et al. 2022) and the FAIR (Findable, Accessible, Interoperable, Reusable) principles, the PSH concept highlights the need for infrastructure that can support both provisional classifications and long-term taxonomic research. The intersection of PSH with DES and FAIR principles underscores the challenges - and critical importance - of establishing, maintaining and scaling digital infrastructure to meet the demands of modern biodiversity research. This is not to argue for a new type of digital infrastructure, but improving on existing infrastructures and aligning global and regional funding schemes that can be adopted to implement such a proposal. Similar to Buckley, Meier et al. (2024) also emphasise that achieving integrative taxonomy (combining morphological, whole organism study with molecular data) requires reliable data handling, including efficient voucher storage, standardised data practices and FAIR-compliant infrastructure to support the evolution of taxonomic hypotheses as new data are added.

For biodiversity data to be effective, including taxonomic and nomenclature information, a resilient infrastructure is crucial to maintain links amongst evolving species hypotheses, underlying specimens, environmental observations and genetic data. Efforts to create such infrastructures have accelerated globally as we confront biodiversity and climate crises (Devictor and Bensaude-Vincent 2016). Although global data infrastructures that support biodiversity data and research funding are unevenly distributed, the DES and PSH approach could mitigate disparities by providing an inclusive, interoperable system that enables biodiversity data sharing across regions and disciplines.

The DES, as proposed, is a paradigm for digitally linking specimen data from global natural science collections to related taxonomic, ecological and environmental data. DES enables the transformation of physical specimen data into digital objects, making them accessible and FAIR. This approach not only broadens usability, but also enhances the value of collections by integrating them into global data infrastructures that can be leveraged for large-scale, multifactor analysis (Heberling et al. 2021). Thinking about DES, PSH and FAIR in a holistic framework brings up the notion of pluralistic data pooling advocated by Sterner et al. (2023):2:

We define ‘data pooling’ for biodiversity data as a process that combines data from multiple sources into one taxonomically standardized body of information, provides infrastructure for managing and accessing the combined data and governs it as a shared resource for a community of users and stakeholders beyond a single research project or lab. We define ‘taxonomic standardization’ as a set of processes for verifying and re-identifying a collection of species observations as needed to ensure that they are classified in a standardized way according to a single, coherent taxonomy of choice. More generally, ‘data standardization’ (also known as data harmonization) is an established term in academic and industry data science practices.

Part of this set of process can be a PSH data element that can accommodate evolving taxonomic concepts, while ensuring reliable links between data sources. It allows for both the robustness of Linnean taxonomy and the flexibility of documenting hypotheses, thereby fostering a dynamic approach to biodiversity research. Echoing Sterner (also Leonelli (2020) and Borgman and Wofford (2021)), the challenges of biodiversity data collection, sharing and preservation are as much social as technical, thus:

“…making biodiversity data comprehensively available and reusable will likely require major changes to the cultures, organizations and infrastructures of the research communities involved” (Sterner et al. 2023: 2).

This also brings up the notion of maintenance and support. As Borgman et al. (2016) note, "durability" in infrastructure requires continuous maintenance across technical and human resources. Applying this insight to biodiversity data infrastructure highlights that building a sustainable, FAIR-compliant system requires not only technical innovation, but also governance and investment. Borgman’s work in astronomy shows that even well-established systems still face fragility without regular support - an important reminder as we build infrastructures that will support biodiversity data on a global scale.

## Integration with Global Data Standards and Networks

As mentioned already, PSH can expand beyond New Zealand and entomology; it has potential for integration with global biodiversity data initiatives. Organisations and platforms such as the Catalogue of Life, GBIF, BCON, ALA, INSDC, BOLD, UNITE and DiSSCo provide frameworks, tools and services for aggregating and curating biodiversity data, which could be expanded to incorporate PSH as a new type of digital object. By embedding provisional species data into the global biodiversity network, PSH could become widely accessible and actionable across regions and disciplines.

As Moersberger et al. (2024) emphasise in their study on European biodiversity monitoring, integrating biodiversity data is crucial for reducing fragmentation and filling taxonomic gaps. Aligning PSH with the shift toward digital taxonomy could further bridge the divide between morphological and molecular approaches, providing traceable, reusable links to each hypothesis’s provenance. This would enable a more cohesive and adaptable taxonomy, supporting dynamic updates as new data become available.

## Enhancing PSH with FAIR Compliance

**Figure 1.**
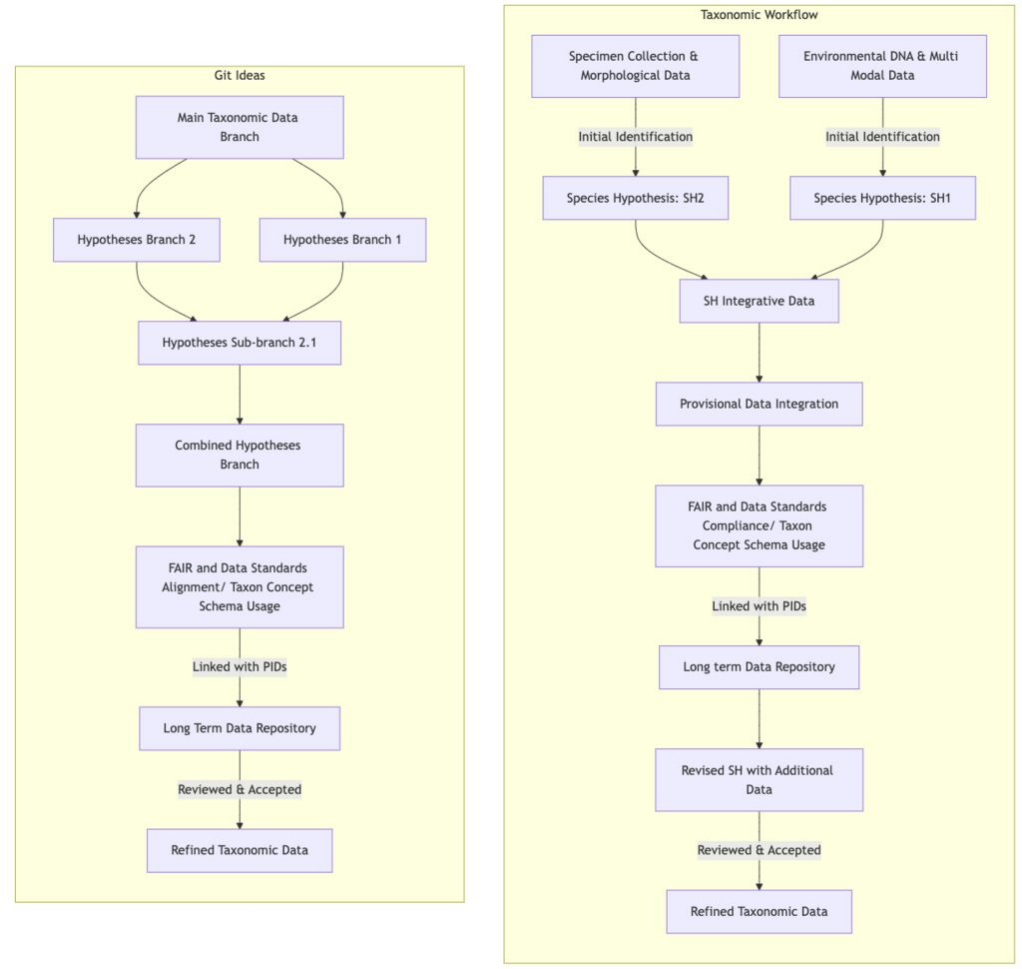
A simplified conceptual framework for version-controlled taxonomic data management This diagram illustrates the parallel between hypothesis-driven taxonomic workflows and Git-based version control systems. Drawing inspiration from software development practices, the framework demonstrates how version control concepts could be applied to manage and track the evolution of taxonomic hypotheses. The actual processes involved are much more complex, as described in Pyle's paper "*An Introduction to Scientific Names of Organisms and the Taxon Concepts they Represent* (Pyle 2022).

To fully realise PSH, we need infrastructure that is both accessible and FAIR-compliant. These hypotheses will function as data points or nodes within a knowledge graph (Page 2019,Penev et al. 2024) and, because they could be stored across multiple infrastructures (Sterner et al. 2020), data linking and interoperability are essential. The Upham and Poelen (2024) concept of Taxonomic Data Objects aligns with this need by offering machine-readable digital packages that encode metadata, enabling the tracking of evolving species concepts over time. Initial taxonomic data can also be compared to a software commit in Git: each PSH represents a specific "state" of species classification, preserving the evolution of taxonomic understanding without overwriting earlier hypotheses. This approach provides a clear pathway for reviewing and merging provisional classifications with established taxonomies, strengthening taxonomic workflows by ensuring data integrity and interoperability across different taxonomic systems (see Fig. 1 for a simple schematic comparing Git merging with the process described using PSH).

## Practical Requirements for Preliminary Species Hypotheses Implementation

For PSH to serve as a valuable tool in taxonomy and biodiversity informatics, certain key elements are essential. This is an initial proposal and will benefit from further discussion:

1. **Persistent Identifiers (PIDs)**: Each PSH digital object should be assigned a PID to ensure reliable tracking and referencing, similar to the approach used for Digital Extended Specimens within the FAIR Digital Object framework (Islam et al. 2023). As suggested by Upham and Poelen (Upham and Poelen 2024), versioning and hashing could be incorporated as part of the metadata to support tracking changes over time. Assigning PIDs to taxonomic data and hypotheses is not a new concept; for example, the Catalogue of Life assigns identifiers for name usage and checklists (Bánki et al. 2023) and UNITE assigns DOIs to species hypotheses (Kõljalg et al. 2020). The discussion should not focus on which specific PID mechanism is optimal - though implementation details are important - but rather on establishing a consensus and actionable plan to assign PIDs to these entities at a granular level. This will enable effective tracking and linking, but requiring dedicated infrastructure and ongoing maintenance support. By assigning transparent and persistent identifiers to contributors across all stages of a species hypothesis’ evolution, the framework could foster equitable recognition while maintaining rigorous standards for formal naming.
2. **Interoperable Data Standards**: Standards like Darwin Core and Taxon Concept Schema (TCS) are necessary to harmonise species hypothesis data with other biodiversity data types, such as observation and occurrence data. Consistent standards enable smoother integration and reuse of taxonomic information across platforms. How a preliminary concept could be part of Darwin Core and other standards framework will need careful consideration. For instance, “dwc:previousIdentifications” property in Darwin Core could store the reference to preliminary data . PSH, SH and TH could have their own data model and metadata, but this also needs global consensus. As new data and insights are being generated, standards and schemas are essential for usability in diverse contexts. While Darwin Core is widely used, TCS’s separation of Taxon Concepts from Taxon Names allows greater flexibility for mapping and resolving taxonomic data. TCS could possibly accommodate dynamic states such as "Preliminary" and "Final" as new insights emerge. It could also address provenance and attribution, akin to the Linnaean tradition of authorship, requiring each state to have a source ("accordingTo") (Klazenga and Liljeblad 2024).
3. **FAIR Principles**: Along with PIDs, machine-readable formats and data standards will enhance accessibility, interoperability and reusability, supporting transparent and evolving taxonomic classifications. Similar ideas have been proposed by Miralles et al. (2020) in the context of alpha taxonomy repositories. Taxonomic Data Objects (Upham and Poelen 2024) could standardise PSH data in a machine-readable format, preserving their structure and allowing flexible data use.
4. **Global Coordination and open source practices**: Collaborative efforts with established networks are essential for integrating PSH into a global biodiversity framework. Beyond achieving consensus on metadata standards, the accessibility and publication of these data must remain a priority. Funders, research institutions and collection-holding organisations need to recognise the importance of APIs (Addink et al. 2023), repositories, data stewardship ([Bibr B12223135][Bibr B12223215]) and other foundational infrastructure and commit both human and technological resources to support them. This is especially crucial given that many countries, despite their reliance on biodiversity data for modelling and monitoring, often lack the necessary capacity, expertise or funding to fully exploit its potential (Moersberger et al. 2024). As illustrated by New Zealand's example, where a small population and limited taxonomic expertise hinder the development of comprehensive taxonomic research, many countries depend on international collaboration for taxonomic knowledge. Addressing this taxonomic impediment calls for capacity building, knowledge exchange and the creation of sustainable, FAIR-aligned taxonomic services through coordinated efforts (Buckley 2024). A unified global solution may be impractical, yet stronger coordination in the software and standards that support taxonomic services is critical. This can facilitate the effective use of new data elements like PSH and promote shared governance structures. For instance, the discussions by Sandall et al. (2023) on checklist maintenance can be extended to taxonomic software and service development, where PSH could be tested and refined. Capacity management and funding challenges also require open dialogue, especially given the voluntary nature of many contributions in taxonomy and also in biodiversity informatics and data stewardship. Metrics from open-source projects, such as the "Contributor Absence Factor" (or "Bus Factor") - which assesses how many contributors can be lost before a project is impacted - could help guide efforts towards sustainability. By learning from open-source practices and research software sustainability principles (Cohen et al. 2021), we can enhance taxonomy's resilience and interoperability across regions. While taxonomic expertise remains indispensable, adopting insights from open-source and other data ecosystems will help us to overcome challenges in data infrastructure and interoperability.

## Conclusion

Buckley's concept of PSH, primarily proposed within entomology, parallels existing frameworks like SH in mycology and BINs and OTUs from molecular methods. Despite their overlaps and distinctions, the need for standardised frameworks to manage preliminary and evolving taxonomic data remains crucial. These frameworks address challenges across diverse taxonomic domains, emphasising their potential to create interoperable and dynamic taxonomic practices, but a wider and global discussion is needed to find a holistic solution.

In the context of New Zealand, Buckley advocates for shifting entomological taxonomy away from the primary focus on completing Linnaean classification. Instead, his proposal highlights achievable objectives aligned with realistic funding and timelines, incorporating DNA data and AI methods as preliminary steps towards formal classification. This commentary connects Buckley's proposal to broader initiatives, such as FAIR principles, Digital Extended Specimens, Taxon Concept Schema, Taxonomic Data Objects and open-source software practices. By treating PSH as data points - similar to versioned git "commits" or "preprints" - species identification and classifications can be iteratively refined without losing historical data. This fosters a more adaptable and integrative approach to taxonomy, bridging morphological and molecular data and AI-based identification, while enhancing global biodiversity conservation efforts.
